# 

**Published:** 1898-05

**Authors:** 


### NOTICES.

### Second Tri-State Dental Meeting, Hotel Victory, Put-in-Bay, Lake Erie, Ohio, June 21st, 22d, 23d, 1898.

DEAR SIR:—The Tri-State Dental Meeting is a Union Meeting of the State Dental Societies of Indiana, Michigan and Ohio. Each State Society appoints a Tri-State Committee to represent its own State. This Tri-State Committee in each society selects one of its members to serve on what is known as the Executive Committee. This Executive Committee has entire charge of organizing and conducting the Union Meetings, which are to be held every three years in the different States in rotation. The meetings are of three days' duration—one day being allotted to each State. The President of the State Society presiding on the day allotted to that particular State. The State

Society within whose jurisdiction the meeting is held, becomes Host and furnishes such social entertainment as they may see fit.

These meetings are open to any reputable dentist, his family and friends. There is no routine business, no election of officers, no election to membership, no dues—not a thing for you to do but to discuss and enjoy the program, those present and the entertainment—in short, it will be one time in your life that you have not a thing to worry about, (unless you happen to be on the committee—then 'its different.)

See names and addresses of Executive Committee below. Communications addressed to either will receive prompt attention. Every reputable dentist or dental manufacturer is invited to display himself or his wares.

At the first Tri-State Dental Meeting, held in Detroit, June, 1895, we had about five hundred dentists present. It is said to have been the largest and most enthusiastic meeting held in this country, except the International Congress at Chicago. Indications point to a still larger meeting this year at Put-in-Bay. When you receive the program, you will see an array of talent not often got together at one meeting. Some of it will be entirely new and original, and mind you we will all be together during the whole meeting, so you need not fail in getting all the discussion in and out of the sessions you may desire.

Hotel Victory is selected as the place of meeting for so many reasons that it is impossible to give them all without writing a book—then you would not read any of them, but we will venture to give a few:

1st. The lake ride, in order to get to Put-in-Bay, is a most charming excursion. To those who have enjoyed lake travel, nothing need be said; but to those who have not, it may truthfully be said you have denied yourself one of, and prehaps the finest water trips the world affords. Boats can be taken from Cleveland, Sandusky, Toledo and Detroit. Time cards for the various lines will be sent later on. Will say, however, that there will be from Detroit, a morning boat; from Toledo, morning and afternoon boat; from Sandusky, morning and afternoon boat; from Cleveland the time of sailing has not yet been determined.

As quite a large number will go by Toledo, a headquarters will be established at the Boody House, to be in charge of Dr. Barber, where you can get all accomodations and information regarding your boats, etc. Boody House is near the dock.

2d. Put-in-Bay Island is an historic point. About this island hovers the recollections of a great naval victory, which, through the pluck of Yankee sailors, gave this Nation right of way on the great lakes.

3d. The hotel is one of the finest in the land. Large, airy, splendidly equipped, a dining-room that will seat 1,200 to 1,500, a band furnishes music, a meeting hall that seats 700 to 900, an abundance of committee rooms, ample parlors, bath rooms, in fact all you can ask for at rates from $2.00 to $4.50 per day. More information regarding rates further along.

4th. There are many points of interest to visit. Perry's Cave, National Fish Culture Station, Gibralter, Johnstone's Island, etc., etc.

5th. In the neighborhood of these islands there are more fish caught per year than on any other fishing grounds in the country. You can catch a string if you use the proper bait!

6th. Sailing is good and boats are plenty.

7th. This is an ideal place to bring your wife. When the meeting is held in a city, your wife finds it pretty dull business to sit in a hotel room alone while you attend the sessions, but here they can come and go at will, without escort, visit the various beaches, boat ride, fish, swim, trolley ride, play golf, bicycle ride, chew gum, or indulge in any other athletic sport they may enjoy. There will most likely be some special entertainment provided that they will enjoy. More about that in next letter. Bring all the ladies.

8th. A reputable dentist is supposed to take a bath every summer. By going to Put-in-Bay, this annual function can be attended to with neatness and dispatch.

9th. Last, but not least. The meetings, the exhibits, the living, will be under one roof. We will be ALL TOGETHER ALL THE TIME.

Get up your excursion parties. For instance, the Cincinnati dentists are arranging to go together, via C. H. & D. R. R., personally conducted by President of State Society.

Executive Committee:—Dr. George Hunt,
89 E. Ohio St., Indianapolis, Ind.
Dr. J. Ward House,
Grand Rapids, Michigan.
Dr. J. R. Callahan,
25 Garfield Place, Cincinnati, O.

### Program of the Section on Stomatology of the American Medical Association at Denver, Colo., June 7-10, 1898.

Dr. G. V. I. Brown, Milwaukee, Wis., Chairman's Address; Dr. W. E. Hill, Milwaukee, Wis., Methods of Teaching Materia Medica with Special Reference to the Needs of Dental Students; Dr. G. T. Carpenter, Chicago, Ill., A Method of Handling Alvoelar Pyorrhoea; Dr. W. H. Hall, Denver, Colo., Only a Baby Tooth; Dr. J. Taft, Cincinnati, Ohio, Management of Pulpless Teeth; Dr. V. A. Latham, Chicago, Ills., Dental Septicemia of the Antrum; Dr. L. G. Noel, Nashville, Tenn.; Dr. A. H. Thompson, Topeka, Kansas, Jaw Movements in Relation to Tooth Forms; Dr. W. Knight, Cincinnati, Ohio, Epulides; Dr. Eugene S. Talbot, Chicago, Ills., Irregularities of the Dental Arch; Dr. A. E. Baldwin, Chicago, Ills., Some Facial Deformities and their Prevention; Dr. V. A. Gudex, Milwaukee, Wis., The Resistance of the Mucous Membranes to Bacterial Infection; Dr. A. H. Sawins, Denver, Colo., The Morbid Susceptibility of Dental Structures is Greater than that of Other Tissues; Dr. J. M. Porter, Denver, Col., Have We Progressed?

Dr. G. V. I. Brown, Chairman.
Dr. Eugene S. Talbot, Secretary.

### Editorial.

### American Medical Association—Dental Section.

The annual meeting of the American Medical Association is to be held in Denver, Colorado, June 7 to 12, 1898.

This will be of special interest to dentists, from the fact that there is a section of that body on Dental and Oral Surgery. This section was organized about fifteen years ago, and has had its meetings in connection with the Medical Association annually. They have always been interesting and elicited much that was valuable.

A good programme has been prepared which will be seen on another page of this issue of the REGISTER. Another interesting feature will be the holding of the Colorado State Dental Society jointly with this section, and a very enjoyable time will be had.

This will afford an opportunitty to see the West, or certain portions of it at least, that does not often occur.

The rate will be one-half fare, with $2 additional. Stop-overs will be given on tickets reading via Omaha, in order that the holders may attend the Omaha Exposition.

On going, trip stop-overs will be allowed up to the last date of sale. On return trip stop-overs will be allowed at any time within return transit limit. Tickets are to be deposited with the agent at Omaha and taken out of deposit at any time within ten days of date of deposit.

There will probably be a through train service from Chicago to Denver, and possibly from Cincinnati.

This meeting is to be held at about the most pleasant time in the year, and undoubtedly this will be a delightful trip, well repaying for time and money in the pleasure afforded, as well as the benefit to be derived from the meetings.

It is to be hoped that all who can make it convenient will go. Members can take their wives or members of their family at the reduced rates.

This trip is to be via the Union Pacific which is a pleasant route and the road one of the best equipped and conducted in the country, one over which it is a pleasure to travel.

Time from Chicago to Denver about twenty-eight hours. The round trip rate for this excursion is $37.50 from Cincinnati.

Further information can be had by addressing A. G. Shearman, Gen. Passr. Agent, Cincinnati, Ohio. Room 35, Carew Building.

Dr. H. T. Whitney, a native of Ohio and president of the China Medical Missionary Association, is now engaged in the difficult work at his home in China in translating Gray's Anatomy into Chinese.

### CONTENTS.

### THE DENTAL REGISTER,

A MONTHLY MAGAZINE DEVOTED TO THE INTERESTS OF THE

**DENTAL PROFESSION.**

*Terms Reduced to* $2.00 *Per Year. Single Copies,* 25 *Cents.*

**EDITED BY PROF. J. TAFT, D.D.S.**

—AND—

**Published by SAM'L A. CROCKER & CO., Ohio Dental and Surgical Depot.**

The volume commences in January and closes in December.

The REGISTER being issued monthly is always fresh, therefore Indispensable to the practitioner who desires to keep posted up with the new inventions and improvements which are daily developed. Neither expense nor effort will be spared to give value and variety to its contents Articles will be illustrated with well executed engravings whenever they will add to the interest or assist to explanation.

Its extensive circulation and frequency of issue make it one of the BEST DENTAL ADVERTISING MEDIUMS in the United States.

All subscriptions must begin with January or July.

Local or special notices, five lines or less, will be inserted for $3 each insertion ; over five lines, 50 cts. per line.

All advertising bills payable in advance, or at furthest, after the issue of the first number containing the advertisement. All transient advertisements to be paid for in advance.

☞CONTRIBUTIONS SOLICITED.

Exclusively Editorial Communications should be addressed to the Editor.

The latest number of the REGISTER may be ordered with or without other good[illegible] any time. and all letters should be addre[illegible]

